# Acute, chronic, and genotoxic studies on the protopine total alkaloids of the *Macleaya cordata* (willd.) R. Br. in rodents

**DOI:** 10.3389/fphar.2022.987800

**Published:** 2022-09-28

**Authors:** Zhen Dong, Shu-sheng Tang, Xiao-lan Ma, Bin Tan, Zhao-shan Tang, Chang-hong Li, Zi-hui Yang, Jian-guo Zeng

**Affiliations:** ^1^ College of Veterinary Medicine, Hunan Agricultural University, Changsha, China; ^2^ Key Laboratory of Chinese Veterinary Medicine in Hunan Province, Hunan Agricultural University, Changsha, China; ^3^ College of Veterinary Medicine, China Agricultural University, Beijing, China; ^4^ College of Veterinary Medicine, Shanxi Agricultural University, Jinzhong, China; ^5^ Hunan MICOLTA Biological Resources Co.,Ltd, Changsha, China

**Keywords:** Macleaya cordata (willd.) R. Br., protopine alkaloids, acute toxicity, chronic toxicity, mutagenicity, teratogenicity, reproductive toxicity, embryonic developmental toxicity

## Abstract

The protopine alkaloids are widely distributed within the opium poppy family and have a wide range of pharmacological effects. MPTA is a product of the protopine total alkaloids extracted from the *Macleaya cordata* (Willd.) R. Br. Previously, we reported good anti-inflammatory activity of MPTA as well as oral acute and sub-chronic toxicity studies in rats. In order to perform a systematic toxicological safety assessment of MPTA, oral acute toxicity, genotoxicity (bone marrow cell chromosome aberration test, sperm abnormality test, bone marrow cell micronucleus test, and rat teratogenicity test), and chronic toxicity in mice were performed in this study. In the oral acute toxicity test, the LD_50_ in ICR mice was 481.99 mg/kg, with 95% confidence limits ranging from 404.27 to 574.70 mg/kg. All three mutagenicity tests tested negative in the range of 60.25–241.00 mg/kg. The results of the teratogenicity test in rats showed no reproductive or embryonic developmental toxicity at only 7.53 mg/kg, which can be considered as a no observed effect level (NOEL) for the teratogenicity test. Therefore, MPTA is safe for use at the doses tested, but attention should be paid to the potential risk to pregnant animals and the safety evaluation and toxicity mechanisms in target animals should be further investigated.

## Introduction

Protopine alkaloids (PAs), represented by protopine (PRO) and allocryptopine (ALL), are a class of berberine ring-opening compounds containing carbonyl groups, which are widely distributed within the poppy family, such as *Macleaya cordata* (Willd.) R. Br., *Hypecoum erectum* L., *Eomecon chionantha* Hance, and *Corydalisyanhusuo* W.T. Wang, which is often used in traditional Chinese medicine (Cai et al., 2020; Lu et al., 2020; Xu et al., 2021; Yuan et al., 2021). There is abundant evidence that PRO is one of the material bases for the traditional effects of *Corydalisyanhusuo* W.T. Wang, namely promoting the flow of qi and blood and relieving pain. PRO and ALL were also considered to be quality markers for Yuanhu Zhitong tablets, an herbal formula containing *Corydalisyanhusuo* W.T. Wang (Li et al., 2018). Modern pharmacological studies have shown that PRO has anti-platelet agglutination and antithrombotic effects, and inhibits both phospholipase and thromboxane synthase (Shiomoto et al., 1990, 1991; Saeed et al., 1997); PRO can exert analgesic effects by modulating dopamine receptor D2 (DRD2) mRNA expression and inhibiting voltage-gated sodium channels (Xu et al., 2021; Zhang et al., 2021); PRO and ALL have also been reported to have potential anti-inflammatory, anti-cancer, antioxidant and anti-arrhythmic activities (Fu et al., 2016; Alam et al., 2019; Nigdelioglu Dolanbay et al., 2021; Yuan et al., 2021). Previously, we reported that protopine total alkaloids (MPTA), isolated from the acidic waste stream used for the extraction of the benzophenanthridine alkaloids in *Macleaya cordata* (*M. cordata*), showed good inhibitory effects on carrageenan and xylene-induced inflammatory edema in rats (Dong et al., 2022b). We have also experimented with the use of MPTA in laying hens to improve intestinal health and egg quality (Liu et al., 2022).

Although PAs exhibit good pharmacological activity, little information on their toxicology has been reported. To date, only one study has reported oral acute toxicity results in mice for PRO isolated from *M. cordata* (Hu et al., 2021). In that study, PRO was considered to be the most toxic compound in *M. cordata*, with an oral LD_50_ of 313.10 mg/kg in mice. Our laboratory previously conducted oral acute toxicity and 90-days subchronic toxicity studies of MPTA in SD rats. The oral LD_50_ of MPTA for rats was 481.99 mg/kg, and at the dose tested, MPTA showed no significant subchronic toxicity, with a no observed effect level (NOEL) considered to be 96.40 mg/kg/day (Dong et al., 2022a). Other than that, other toxicological information is still unclear. Therefore, in order to systematically understand the safety of MPTA and to describe the potential systemic and genotoxic risks of MPTA under long-term exposure, this study conducted oral acute toxicity tests in mice, chronic toxicity tests in rats and standardized genotoxicological studies on MPTA with reference to the Chinese Regulations for the Registration of Veterinary Drugs and the Compendium of Technical Guidelines for Veterinary Drug Research to provide support and guidance for further clinical studies on MPTA.

## Materials and methods

### Reagents and test drugs

MPTA (PRO ≥35%, and ALL ≥15%) was supplied by Hunan MICOLTA Biological Resources Co. Cyclophosphamide (CTX) was purchased from Tianjin Chemical Reagent Company (Tianjin, China). Other reagents were purchased from Aladdin (Shanghai, China) and Sinopharm Chemical Reagent Co., Ltd (Shanghai, China).

### Animals

Sprague Dawley (SD) rats and Institute of Cancer Research (ICR) mice were purchased from Beijing Vital River Laboratory Animal Technology Co., Ltd (Beijing, China). The animals were housed in a standard GLP-compliant environment (room temperature: 22–24°C; relative humidity: 55 ± 10%, 12-h artificial light/dark cycle) and given a standard maintenance diet and clean drinking water. All test animals were examined for general physical condition and environmental acclimatization for 7 days. Mice were fasted for 8 h prior to gavage administration of MPTA, but were allowed to drink freely. All animal experiments were approved by the Animal Ethics Committee of China Agricultural University (WTPJ20120022) and strictly followed American Veterinary Medical Association. During the test, test animals with obvious toxicity symptoms or near death were euthanized promptly.

## Experimental design and methods

### Oral acute toxicity test in mice

This part of the test was conducted according to the guidelines for acute toxicity (LD_50_ determination) of veterinary drugs issued by the Ministry of Agriculture and Rural Affairs of the People’s Republic of China (MARA, PRC) (Ministry of Agriculture and Rural Affairs of PRC, 2009d). Based on the results of the pre-experiments, the doses were determined as 1000.00, 666.67, 444.44, 296.30 and 197.53 mg/kg. Fifty ICR mice, weighing 18–22 g, were randomly divided into five groups of 10 mice each, with five males and five females in each group. Mice were gavaged with 0.2 ml of suspension per 10 g body weight (i.g). General status observations, signs of intoxication and mortality were monitored and recorded at 30 min, 1, 2, 4 and 8 h after drug administration. The animals were observed twice a day for 7 days. All animals that died during the observation period were subjected to necropsy.

### Sperm abnormality test in mice

The sperm aberration test refers to the guidelines for sperm abnormality tests in mice of veterinary drugs of MARA, PRC (Ministry of Agriculture and Rural Affairs of PRC, 2009c). Fifty male ICR mice (25–35 g) were randomly divided into five groups of 10 mice each. The assigned mice were administered MPTA at 60.25 mg/kg, 120.50 mg/kg, and 241.00 mg/kg for five consecutive days, respectively. Distilled water and CTX (40 mg/kg) were administered to the negative control and positive control groups, respectively. On day 35 after the first dose, five mice were randomly selected and executed in each test group. Selected mice were removed twice from the epididymis and smears of sperm specimens were made in a standard procedure that could be used for observation (Wang et al., 2015). Sperm morphology was examined under a microscope, and normal and various types of abnormal sperms under the same field of view were counted and recorded (Anwar et al., 2022).

### Mouse bone marrow cell micronucleus test

The micronucleus test was designed using the guidelines for bone marrow cell micronucleus test in mice of veterinary drugs of MARA, PRC (Ministry of Agriculture and Rural Affairs of PRC, 2009a). One hundred ICR mice (25–30 g) were randomly divided into five groups of 20 mice each, with 10 males and 10 females in each group. The doses administered to each group of mice were 60.25 mg/kg, 120.50 mg/kg and 241.00 mg/kg, respectively, and two doses were administered, with the second dose administered 24 h after the first dose. Mice in the negative control group were administrated the same volume of distilled water, and the positive control group was administrated 40 mg/kg CTX. The mice were executed 6 h after the second gavage, and the femur was removed from both sides of each mouse, the muscle was shaved off, the surface blood was wiped off with filter paper, and both ends of the femur were cut off. The bone marrow cavity was then flushed several times with about 0.5 ml of fetal bovine serum using a 1 ml syringe equipped with a No. Six needle, and the bone marrow flush solution was mixed to make smears. Smears were fixed in methanol for 15 min, and stained with Giemsa dye for 15 min and observed under oil microscope for normochromatic erythrocyte (NCE) and polychromatic erythrocytes (PCE). Count the number of PCEs containing micronuclei in 1000 PCEs and count the ratio of PCE to NCE in 200 cells.

### Mouse bone marrow cell chromosome aberration test

The chromosome aberration test was conducted in accordance with the guidelines for bone marrow cell chromosome aberration test in mice of veterinary drugs of MARA, PRC (Ministry of Agriculture and Rural Affairs of PRC, 2009e). Eighty ICR mice (25–30 g) were randomly divided into five groups of 16 mice each, half being females and the other half being males. Three dose groups (60.25 mg/kg, 120.50 mg/kg and 241.00 mg/kg) were set up and administered three times at 24 h intervals. CTX (40 mg/kg) was used as a positive control and distilled water as a negative control. Twenty-4 hours after the last dose, five mice from each group were randomly selected for humane execution. 4 h before execution, the animals were injected intraperitoneally with 0.04% colchicine (4 mg/kg). Using 5 ml of 2.2% sodium citrate solution, bone marrow from the femur was washed into a 10 ml centrifuge tube. The bone marrow was suspended by pipetting, then centrifuged at 1000 r/min for 10 min, and the supernatant was discarded to collect the precipitated bone marrow cells. Bone marrow cells were fixed using methanol: acetic acid (3:1), and stained with Giemsa solution for 15 min 100 metaphase cells were observed in each animal, and cells with abnormal chromosome structure and number were observed and recorded.

### Traditional teratogenicity test in rats

The teratogenicity test followed the guidelines for classical teratogenicity tests in rats of veterinary drugs of MARA, PRC (Ministry of Agriculture and Rural Affairs of PRC, 2009b). Eighty uncrossed young females (220–250 g) and 40 sexually mature males (250–300 g) of SD rats were used for teratogenicity tests. Based on the acute toxicity results, three dose groups of 7.53 mg/kg, 30.12 mg/kg and 120.50 mg/kg, and a negative control group (distilled water) were set up with 20 female and 10 male rats in each group. Two female and one male rats were assigned to each cage for mating. Mating success was determined by checking whether sperm could be observed in the vaginal smear of female rats, and the day was taken as day 0 of pregnancy, and continued until 12 pregnant rats were reached in each group. Mated female rats were housed individually in transparent polycarbonate cages and were allowed to feed and drink freely. All pregnant rats were administered by gavage once daily from day 7 to day 16 of gestation. Pregnant rats were weighed on days 0, 7, 12, 16 and 20 of conception, and observed daily for general behavior, intoxication and death, and in case of death, post-mortem examinations were performed. All pregnant rats were executed at day 20 of gestation, ovaries and uterus were removed and examined for the number of corpus luteum, number of absorbed fetuses, number of stillbirths, number of live fetuses, male to female ratio in live fetuses, position of the live fetuses in the uterus and weight of ovaries, uterus and uterus attached to the fetus (including amniotic fluid). Afterwards, the weight, body length and tail length of the live fetuses were measured and the live fetuses were examined for cosmetic deformities.

Half of the live fetuses from each litter were fixed by ethanol for 2–3 weeks. The fixed specimens were rinsed in running water and put into potassium hydroxide solution transparently for 72 h, and then put into alizarin red S staining solution (2%, pH = 4.2) for staining for 48 h, and shaken lightly 1–2 times a day. After the skull was stained red, the fetal mouse specimens were removed and placed in two different concentrations of glycerol-potassium hydroxide solution transparently, and removed after all the skeletal specimens were stained red and the soft tissue was discolored. The fetal specimens were observed under a transmission light source using a stereomicroscope, and the number of sternums, incomplete ossification, rib and limb bone abnormalities, and spinal development (fusion or longitudinal cleft) were examined and recorded in each test group. The other half of the live fetus was placed in Bouin’s fixative solution and the fetal viscera were examined after 2 weeks of fixation, and the size, shape and relative position of the organs in different sections of the fetal rat were observed and recorded for abnormalities (Anwar et al., 2021a; 2021b).

### Chronic toxicity test in rats

Two hundred SD rats (70–90 g) were randomly divided into four groups including three dose groups (48.20 mg/kg, 9.64 mg/kg and 1.93 mg/kg) and one negative control group (no addition). MPTA was added to the diets at each dose [daily dietary intake of rats was calculated as 8% (1–90 days) and 6% (91–180 days) of body weight, respectively)] and given to the test rats via *ad libitum* feeding. During the feeding test, the behavior, mental status, food and water intake, toxicity and death of the rats were observed and recorded daily. The body weight, food intake and water intake of all rats were measured every 5 days.

On days 45, 90, 135 and 180 of the experiment, 10 rats in each group (five males and five females) were randomly selected and anesthetized by intraperitoneal injection of urethane (ethyl carbamate). Blood samples were obtained by cardiac puncture for further hematological examinations such as red blood cell count (RBC), white blood cell count (WBC), platelet count (PLT) and hemoglobin (HGB), as well as alanine aminotransferase (ALT), aspartate aminotransferase (AST), total protein (TP), albumin (ALB), creatinine (CR), blood urea nitrogen (BUN), glucose (GLU) and total cholesterol (TCH). The rats were dissected for suspicious histological lesions, and the major organs (heart, liver, spleen, lungs, kidneys, gastrointestines, testes and ovaries) were weighed and the corresponding organ coefficients were calculated. Organ coefficient = organ weight/body weight. At the end of the test, histopathological examinations were performed on the major organs of rats in the 48.20 mg/kg and control groups, as well as on suspicious lesions observed during gross autopsy, and on rats in the lower dose group if toxicity-related lesions were found.

### Statistical analysis

Appropriate statistical analysis of the data was performed using SPSS 26.0 (International Business Machines Corporation, Armonk, USA) with *t*-test and one-way ANOVA for body weight, feed intake, water intake, hematology, and clinical chemistry results; Percentages were tested using two tests. Image production was performed by GraphPad Prism 9.0.0 (GraphPad Software, San Diego, USA). *p*-values less than 0.05 were considered statistically different.

## Results

### Acute toxicity in mice

In acute toxicity tests, death in ICR mice occurred 8–24 h after administration (Table 1), and clinical signs of toxicity were slowed movement and sedentary. Gross autopsy of the dead mice did not reveal any significant pathological changes in the major organs. The oral acute toxicity LD_50_ of MPTA in mice was calculated to be 481.99 mg/kg (95% confidence interval 404.24–574.70 mg/kg) based on the modified Kärber’s method (Mantel, 1967).

**TABLE 1:** Oral acute toxicity of MPTA to mice (n = 10).

Dose group (mg/kg)	Number of animals	Number of deaths	Mortality rate (%)	Survival rate (%)
1000.00	10	10	100	0
666.67	10	8	80	20
444.44	10	4	40	60
296.30	10	1	10	90
197.53	10	0	0	100

### Sperm abnormality test in mice

In the sperm aberration test, the positive drug, CTX, significantly increased the number of aberrations and the aberration rate of mouse sperm (*p* < 0.01), in which the proportion of amorphous sperm increased and the proportion of banana-shaped, double-tailed sperm decreased. Compared with the negative control group, MPTA at 120.50 mg/kg increased the number of no-hook sperm; all three dose groups of MPTA and CTX decreased the number of banana-shaped sperm; except for CTX which significantly increased the number of amorphous sperm, none of the MPTA treatment groups were significantly different; 241.00 mg/kg, 120.50 mg/kg MPTA and CTX also reduced the number of double-tailed sperm; except for that, only 60.25 mg/kg MPTA increased the number of double-headed sperm. MPTA in the tested range (60.25–241.00 mg/kg) did not cause significant differences (*p* > 0.05) in the total sperm abnormality rate in mice compared to the negative control group, although the different treatment groups had different effects on the type of sperm abnormalities. The results showed that MPTA at doses ranging from 60.25–241.00 mg/kg did not cause sperm malformations in mice (Table 2).

**TABLE 2:** Effect of MPTA on sperm malformation in mice (n = 5).

Groups	Sperm test count	Abnormal sperm count	Sperm abnormality rate	Percentage of each type of abnormal sperm (%)						
				No hook	Banana-shaped	Amorphous	Fathead	Tail-folded	Double-headed	Double-tailed
241.00 mg/kg	5 × 1000	134	2.76 ± 0.84	12.69 ± 0.82	14.93 ± 0.15^**^	33.58 ± 0.47	14.18 ± 0.27^*^	14.93 ± 0.73	3.73 ± 0.65	5.97 ± 0.14^**^
120.50 mg/kg	5 × 1000	141	2.91 ± 0.82	14.89 ± 0.94^*^	14.18 ± 0.59^**^	34.04 ± 0.88	13.48 ± 0.69	12.77 ± 0.79	2.84 ± 0.41	7.80 ± 0.51^*^
60.25 mg/kg	5 × 1000	118	2.42 ± 0.63	10.17 ± 0.50	11.86 ± 0.66^**^	36.44 ± 1.23	13.56 ± 0.52	11.02 ± 0.67	5.93 ± 0.49^*^	11.02 ± 0.75
Negative control	5 × 1000	135	2.78 ± 0.91	11.85 ± 0.70	21.48 ± 1.19	29.63 ± 1.12	10.37 ± 0.73	13.33 ± 0.79	2.96 ± 0.41	10.37 ± 0.73
Positive control	5 × 1000	418	9.12 ± 1.44^**^	9.57 ± 0.97	7.66 ± 1.05^**^	51.20 ± 2.13^**^	11.00 ± 1.49	10.77 ± 1.41	3.83 ± 0.70	5.98 ± 1.07^**^

Note: Data are compared with negative controls, ^*^ indicates significant differences (*p* < 0.05), ^**^ indicates significant differences (*p* < 0.01).

### Mouse bone marrow cell micronucleus test

**FIGURE 1:**
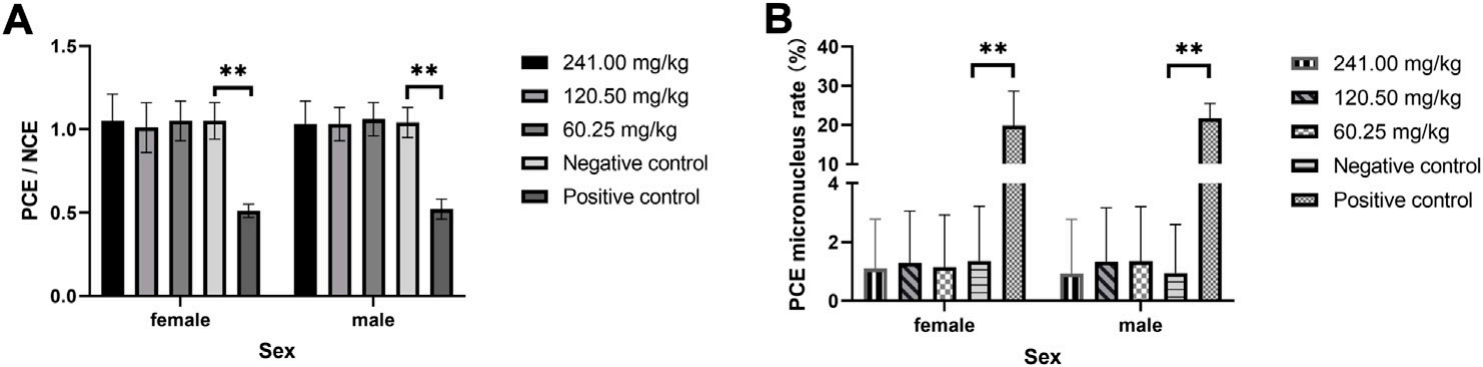
Effect of MPTA on bone marrow micronucleus test in mice (n = 20, females = 10, males = 10) **(A)** Effect of MPTA on PCE/NCE values; **(B)** Effect of MPTA on PCE micronucleus rate. Data are compared with negative controls, and ^*^ indicates significant differences (*p* < 0.05).

The results of the micronucleus test of MPTA in mouse bone marrow cells are shown in Figure 1. The rate of PCE micronucleus in bone marrow cells of female and male mice in the positive control group was significantly higher than that of the corresponding sex in the negative control group (*p* < 0.01). In contrast, the PCE micronucleus rate in both sexes in each dose group of MPTA was not significantly different from that of the negative control group (*p* > 0.05). And the PCE/NCE values also showed the same trend. The results showed that there was no effect on the micronucleus rate of bone marrow cells in the dose range of 60.25–241.00 mg/kg in both males and females.

### Mouse bone marrow cell chromosome aberration test

**FIGURE 2:**
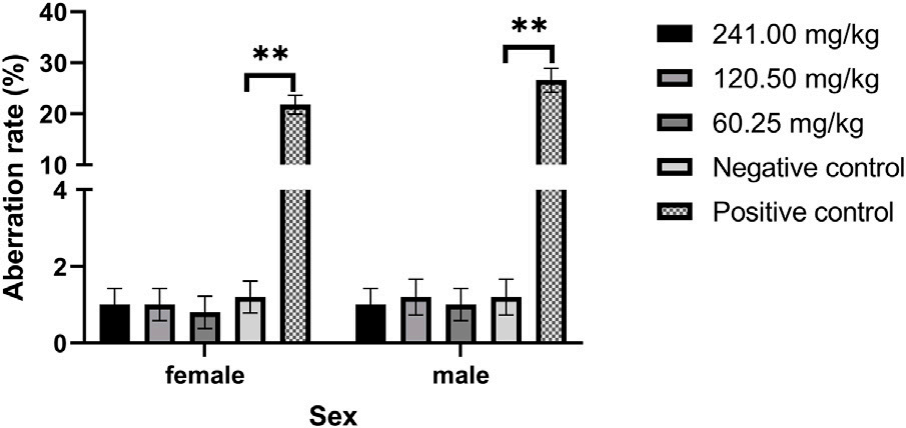
Effect of MPTA on the rate of chromosomal aberrations in mouse bone marrow cells (n = 16, females = 8, males = 8). Data are compared with negative controls, and * indicates significant differences (*p* < 0.05).

As shown in Figure 2, the chromosomal aberration rates of female and male mice cells in each dose group of MPTA (60.25–241.00 mg/kg) were not significant (*p* > 0.05) and significantly lower (*p* < 0.01) than those of the positive control group. The results showed that chromosomal aberrations were not significantly induced in the dose range of 60.25–241.00 mg/kg.

### Traditional teratogenicity test in rats

**FIGURE 3:**
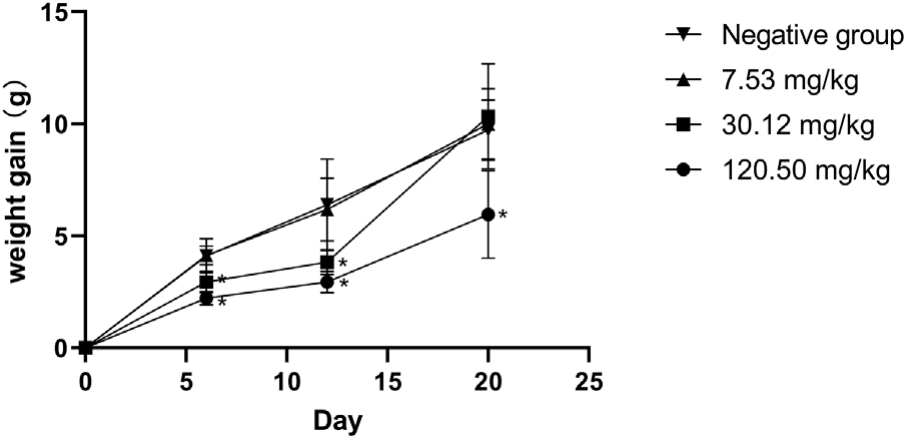
Effect of MPTA on body weight gain in pregnant rats.

During the test period, the pregnant rats in the three MPTA dose groups (7.53 mg/kg, 30.12 mg/kg and 120.50 mg/kg) and the negative control group did not show any poisoning or death, nor did they show any significant abnormalities in diet, water intake and clinical performance. Compared with the control group, the rats in the 120.50 mg/kg and 30.12 mg/kg groups showed significantly delayed weight gain (*p* < 0.05), while the 7.53 mg/kg group showed no significant difference (*p* > 0.05) (Figure 3). The results showed a lack of maternal toxicity in pregnant rats only at 7.53 mg/kg. After execution of the pregnant rats on day 20 of gestation, the indicators related to reproductive function such as ovarian weight, uterine weight, number of corpus luteum, mean fertility rate and mean fetal survival rate in the abdomen of pregnant rats in each test group are shown in Table 3. Further examination of embryos revealed that no stillbirths occurred in any of the four groups, but the embryo absorption rate was significantly higher in the 120.50 mg/kg and 30.12 mg/kg groups compared to the negative control group (*p* < 0.05), and the fetal survival rate was significantly lower (*p* < 0.05) (Table 4). In addition, the placental weight, fetal rat weight and fetal rat body length in the abdomen of pregnant rats were significantly lower (*p* < 0.05) in both 120.50 mg/kg and 30.12 mg/kg groups compared to the negative control group (Table 5), which showed that there were some effects of MPTA on rat fetal development at doses of 120.50 mg/kg-30.12 mg/kg.

**TABLE 3:** Effect of MPTA on reproductive function in rats (n = 12).

Groups	Ovary weight (g)	Uterus weight (g)	Number of corpus luteum	Average number of nidation	Average number of live fetuses
120.50 mg/kg	0.13 ± 0.02	5.77 ± 0.59	13.92 ± 2.35	14.00 ± 1.21	3.67 ± 2.99
30.12 mg/kg	0.14 ± 0.02	5.46 ± 0.68	13.67 ± 1.30	13.17 ± 1.27	11.08 ± 2.19
7.53 mg/kg	0.14 ± 0.02	6.13 ± 0.85	14.92 ± 1.44	13.67 ± 1.56	13.42 ± 1.68
Negative control	0.14 ± 0.02	5.57 ± 0.35	14.00 ± 1.41	13.25 ± 1.86	13.58 ± 1.62

Note: Data are compared with negative controls, and ^*^ indicates significant differences (*p* < 0.05).

**TABLE 4:** Effect of MPTA on the survival rate of rat embryos (n = 12).

Groups	Number of nidation	Number of live fetuses (♀/♂)	Embryo absorption rate (%)	Fetal death rate (%)	Fetal survival rate (%)
120.50 mg/kg	168	21/23	73.81^*^ (124/168)	0 (0/168)	26.19^*^ (44/168)
30.12 mg/kg	158	72/61	15.82^*^ (25/158)	0 (0/158)	84.18^*^ (133/158)
7.53 mg/kg	161	75/82	1.83 (3/164)	0 (3/164)	98.17 (161/164)
Negative control	163	84/75	2.52 (4/159)	0 (0/159)	97.55 (159/163)

Note: Data are compared with negative controls, and ^*^ indicates significant differences (*p* < 0.05).

**TABLE 5:** Effect of MPTA on the development of rat embryos (n = 12).

Groups	Placenta weight (g)	Fetal rat weight (g)	Fetal rat body length (cm)	Fetal rat tail length (cm)
120.50 mg/kg	0.39 ± 0.13*	2.81 ± 0.89*	2.81 ± 0.89*	1.05 ± 0.33
30.12 mg/kg	0.50 ± 0.01	3.51 ± 0.07*	3.29 ± 0.13*	1.21 ± 0.04
7.53 mg/kg	0.52 ± 0.01	3.60 ± 0.05	3.52 ± 0.06	1.21 ± 0.02
Negative control	0.50 ± 0.03	3.64 ± 0.13	3.57 ± 0.09	1.22 ± 0.01

Note: Data are compared with negative controls, and ^*^ indicates significant differences (*p* < 0.05).

**FIGURE 4:**
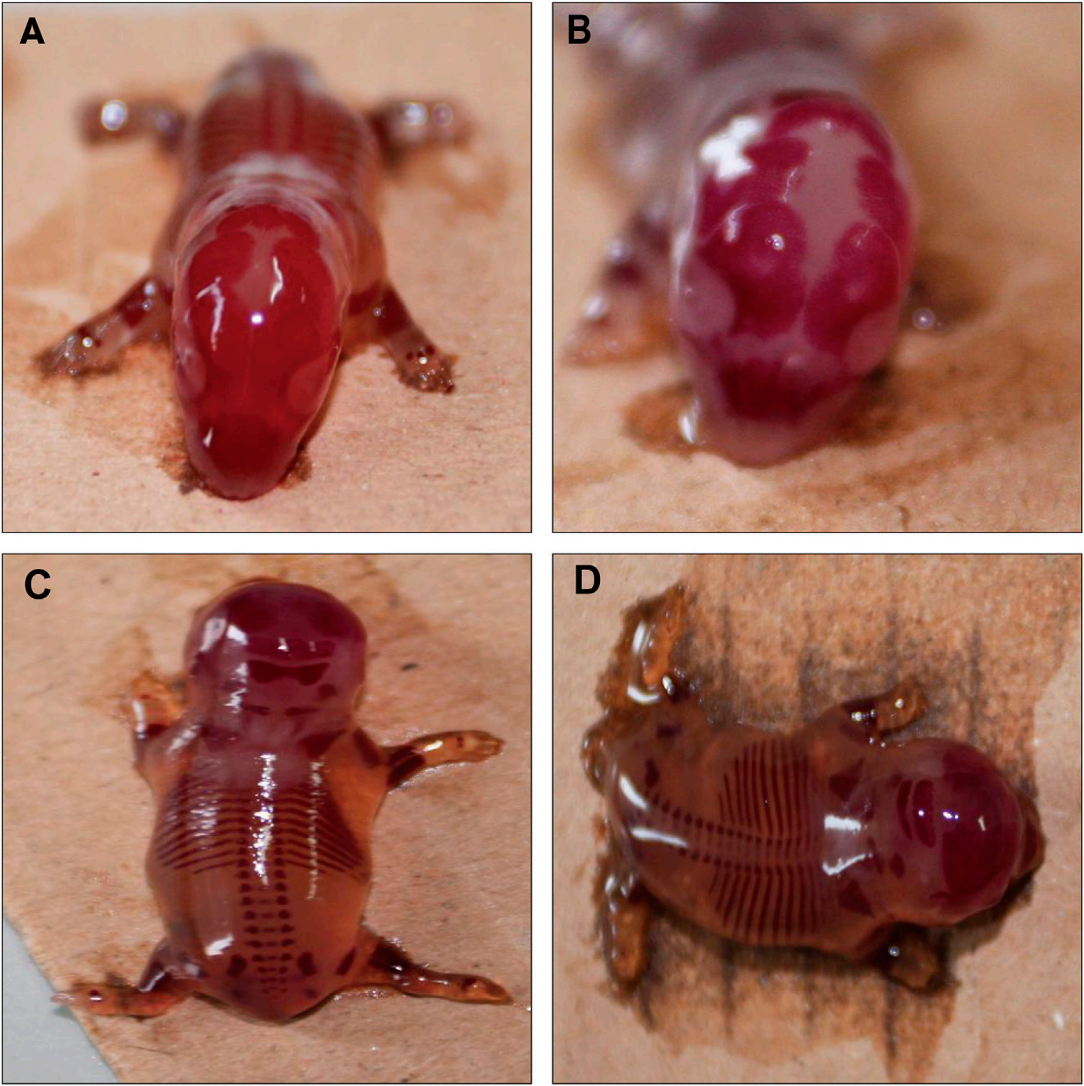
Effect of MPTA on skeletal deformities in fetal rats **(A)** Normal skull; **(B)** delayed skull ossification; **(C)** normal occipital bone; **(D)** delayed occipital ossification.

Table 6 shows the statistical results of MPTA on fetal malformations in appearance, skeletal malformations and visceral malformations in rats. No external and internal malformations were observed in the MPTA dose groups and the negative control group. Retarded ossification (mainly in the skull and occipital bones) was observed in all four groups (Figure 4), with the percentage of fetal rats with retarded ossification in the negative control group within the normal historical control range of the laboratory. By statistical analysis, MPTA induced a higher rate of malformed fetuses and maternal malformations at a dose of 120.50 mg/kg (*p* < 0.05). There was no significant effect of 7.53–30.12 mg/kg on the occurrence of teratogenicity and maternal malformation rate. Therefore, MPTA at doses of 7.53–30.12 mg/kg had no teratogenic effect on the appearance, bones and viscera of fetal rats. In conclusion, MPTA did not affect the feeding, drinking and weight gain of pregnant SD rats at a dose of 7.53 mg/kg, and had no significant effects on the reproductive function, embryonic survival and growth, and the appearance, skeletal and visceral malformations of the fetuses of pregnant rats. Therefore, the NOEL of the MPTA teratogenic test can be considered as 7.53 mg/kg.

**TABLE 6:** Teratogenic effects of MPTA in rats (n = 12).

Groups	Appearance malformations			Skeletal malformations			Visceral malformations		
	Number of fetal mice examined	Teratogenesis rate (%)	Maternal malformation rate (%)	Number of fetal mice examined	Teratogenesis rate (%)	Maternal malformation rate (%)	Number of fetal mice examined	Teratogenesis rate (%)	Maternal malformation rate (%)
120.50 mg/kg	44	0	0	20	80.00* (16/20)	66.67* (8/12)	24	0	0
30.12 mg/kg	133	0	0	55	10.91 (6/55)	33.33 (4/12)	78	0	0
7.53 mg/kg	161	0	0	69	4.35 (3/69)	16.67 (2/12)	92	0	0
Negative control	159	0	0	68	5.88 (4/68)	16.67 (2/12)	91	0	0

Note: Teratogenesis rate (%) = (number of teratology/number of fetal rats examined) × 100%; maternal malformation rate (%) = (number of pregnant rats with teratology/number of pregnant rats examined) × 100%. Data are compared with negative controls, and ^*^ indicates significant differences (*p* < 0.05).

### Chronic toxicity test in rats

**FIGURE 5:**
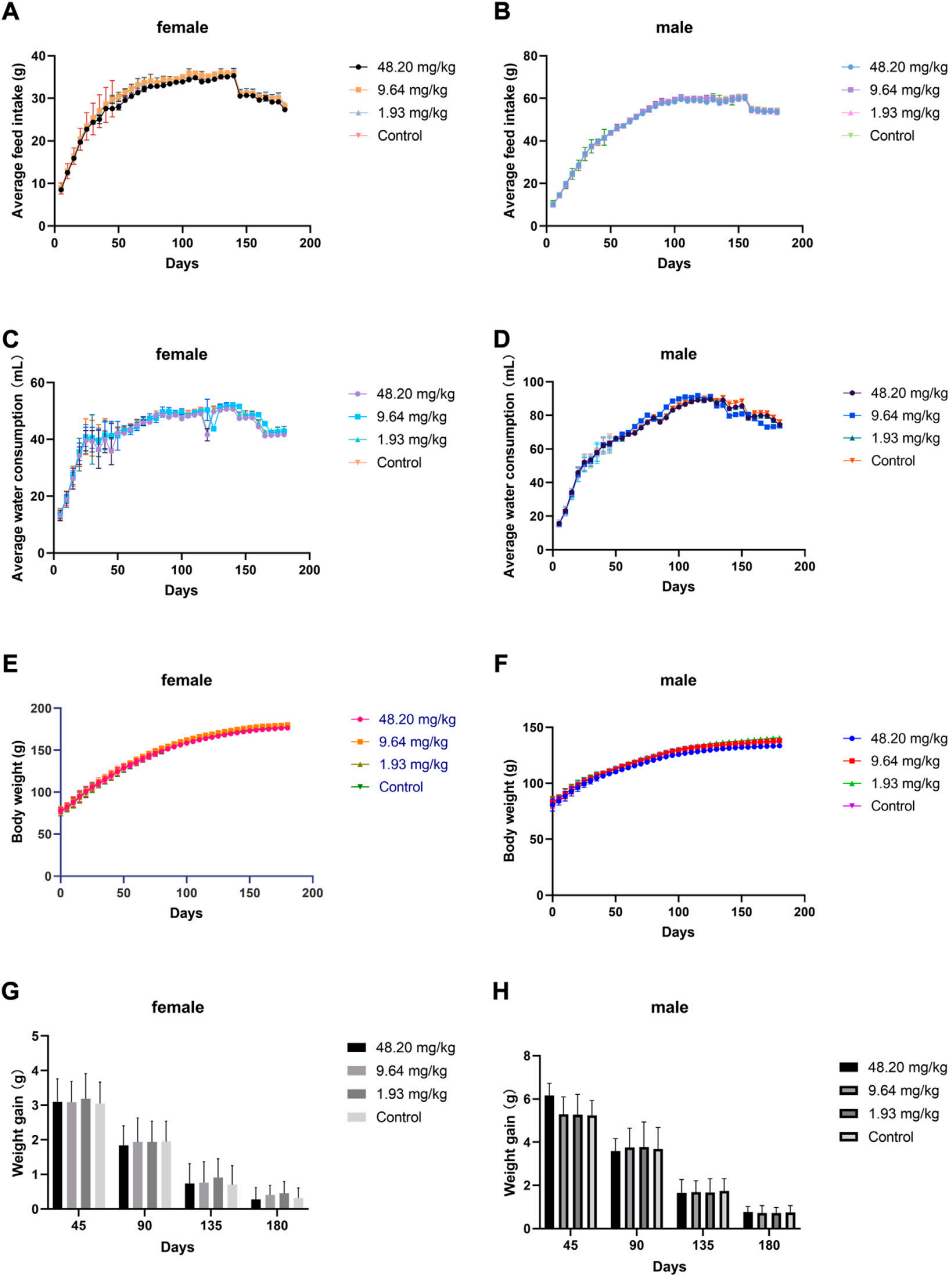
Effects of feeding MPTA for 180 days on feeding, drinking, body weight and weight gain of rats **(A)** Effect of MPTA on feed intake of female rats; **(B)** Effect of MPTA on feed intake of male rats; **(C)** Effect of MPTA on water intake of female rats; **(D)** Effect of MPTA on water intake of male rats; **(E)** Effect of MPTA on body weight of female rats; **(F)** Effect of MPTA on body weight of male rats; **(G)** Effect of MPTA on weight gain of female rats; **(H)** Effect of MPTA on weight gain of female rats.

**FIGURE 6:**
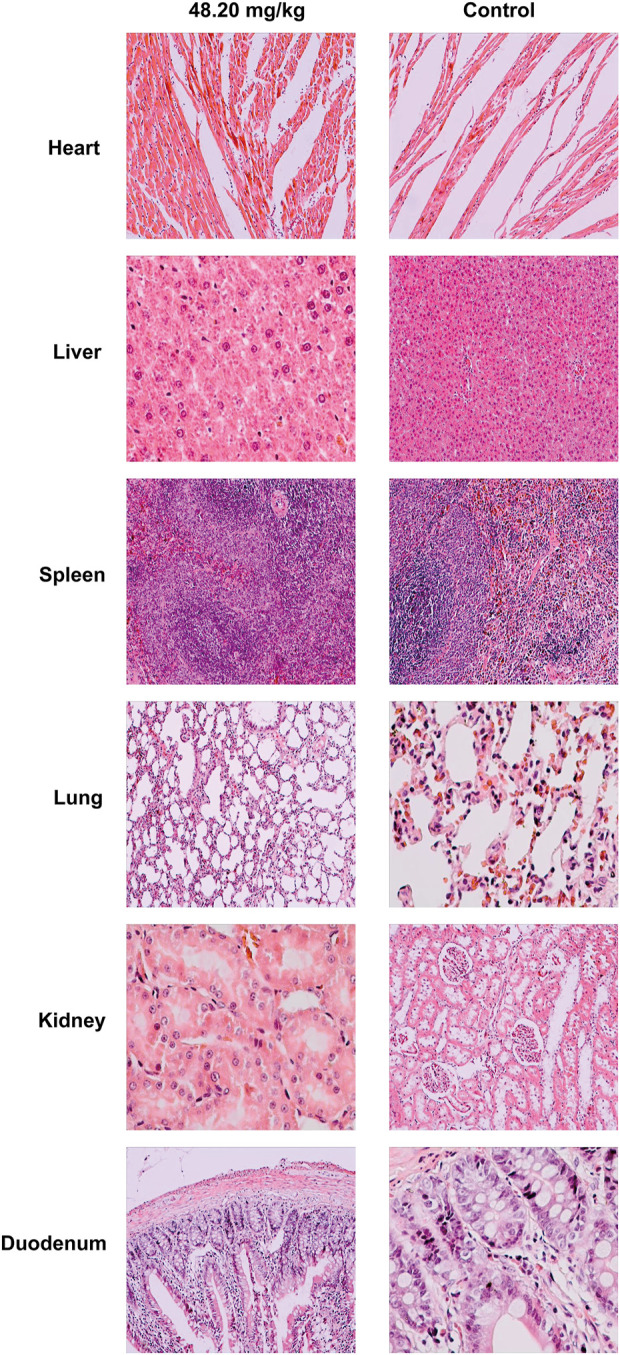
Histopathological effects of MPTA on major organs of rats. Magnification of heart, liver, spleen, kidney in 48.20 mg/kg dose group and heart, spleen, lung, duodenum in control group were ×400; magnification of lung, duodenum in 48.20 mg/kg dose group and liver, kidney in control group were ×200.

During the test period (180 days), animals in the three dose groups (48.20 mg/kg, 9.64 mg/kg and 1.93 mg/kg) and the negative control group of MPTA did not show any abnormal behavior. The feed intake, water consumption, body weight and weight gain of the animals in each test group are shown in Figures 5A–H. There were no significant differences in feed intake, water consumption, body weight and weight gain between the three dose groups of MPTA and the negative control group during the test period (*p* > 0.05). The hematological results of HGB, WBC, RBC, and PLT for each test group at days 45, 90, 135, and 180 of the trial are shown in Table 7. The results of clinical chemistry such as ALT, AST, BUN, CR, GLU, TP, ALB and TCH are shown in Table 8. The hematological and clinical chemistry of the rats in each dose group were not significantly affected from the negative control group (*p* > 0.05). Throughout the test period, no animals showed poisoning and death, and gross autopsy of surviving animals showed no abnormal lesions, and the organ coefficients of rats in each test group were not significantly different from those of the negative control group (Table 9). Further histopathological examination of the rats in the 48.20 mg/kg group and the negative control group showed no significant pathological changes in the major organs of the heart, liver, kidney, spleen, lung, kidney, etc. in both groups as seen in Figure 6. In conclusion, MPTA had no significant effects on feeding, drinking and weight gain of rats in the dose range of 1.93–48.20 mg/kg, and no significant effects on hematological, clinical chemistry and histopathological examinations, and did not show chronic toxicity. Therefore, the NOEL of 180-days chronic toxicity test in rats can be considered as 48.20 mg/kg.

**TABLE 7:** Effect of MPTA on hematological parameters.

Groups	Hematology parameters							
	HGB (g/L)		RBC (M/mm^3^)		WBC (th/mm^3^)		PLT (th/mm^3^)	
	F	M	F	M	F	M	F	M
45 days								
48.20 mg/kg	152.03 ± 7.56	155.80 ± 7.90	7.18 ± 0.58	7.77 ± 0.34	8.11 ± 0.54	11.88 ± 1.32	472.80 ± 65.66	464.00 ± 84.32
9.64 mg/kg	157.80 ± 8.11	154.11 ± 11.01	7.85 ± 0.51	7.41 ± 0.38	8.41 ± 0.62	12.02 ± 1.90	444.00 ± 60.98	435.00 ± 44.89
1.93 mg/kg	164.34 ± 13.48	153.00 ± 5.69	7.56 ± 0.61	7.57 ± 0.35	7.89 ± 0.28	12.97 ± 1.79	435.60 ± 70.52	452.60 ± 54.55
Control	156.40 ± 9.79	150.12 ± 8.52	7.29 ± 0.47	7.23 ± 0.49	8.32 ± 0.61	12.85 ± 0.86	468.50 ± 68.72	454.60 ± 60.88
90 days								
48.20 mg/kg	149.80 ± 7.73	150.20 ± 12.45	7.18 ± 0.58	7.92 ± 0.32	8.11 ± 0.56	9.50 ± 0.64	473.20 ± 65.96	403.40 ± 53.81
9.64 mg/kg	149.90 ± 9.42	151.42 ± 11.70	7.45 ± 0.47	7.86 ± 0.38	7.58 ± 0.85	9.76 ± 1.16	377.20 ± 59.87	379.80 ± 69.00
1.93 mg/kg	154.20 ± 8.45	150.60 ± 8.62	7.45 ± 0.49	7.89 ± 0.37	7.56 ± 0.52	10.22 ± 0.82	400.00 ± 52.51	376.00 ± 56.98
Control	147.40 ± 10.09	153.42 ± 10.18	7.31 ± 0.52	7.66 ± 0.52	7.10 ± 0.47	9.84 ± 0.58	373.00 ± 66.02	361.80 ± 60.45
135 days								
48.20 mg/kg	149.60 ± 7.76	157.35 ± 6.24	7.24 ± 0.57	7.77 ± 0.32	8.15 ± 0.57	8.36 ± 0.85	472.00 ± 66.21	461.60 ± 85.46
9.64 mg/kg	155.40 ± 7.61	156.31 ± 21.75	7.47 ± 0.61	7.43 ± 0.43	8.47 ± 0.67	8.04 ± 0.79	448.20 ± 61.47	439.60 ± 42.19
1.93 mg/kg	156.98 ± 12.58	156.80 ± 6.94	7.63 ± 0.59	7.53 ± 0.47	7.97 ± 0.28	8.59 ± 0.43	440.60 ± 71.21	456.40 ± 54.80
Control	154.20 ± 10.09	154.72 ± 7.77	7.25 ± 0.49	7.19 ± 0.49	8.28 ± 0.62	7.97 ± 0.86	469.40 ± 69.51	459.60 ± 58.08
180 days								
48.20 mg/kg	149.60 ± 7.76	149.80 ± 7.57	7.24 ± 0.57	8.31 ± 0.75	8.15 ± 0.57	7.97 ± 0.45	472.00 ± 66.21	403.00 ± 54.86
9.64 mg/kg	149.70 ± 8.47	153.22 ± 11.86	7.48 ± 0.48	7.79 ± 0.37	7.76 ± 0.50	7.94 ± 1.14	377.00 ± 58.89	381.60 ± 68.05
1.93 mg/kg	154.00 ± 9.40	150.80 ± 9.24	7.51 ± 0.51	7.81 ± 0.37	7.60 ± 0.54	8.23 ± 0.82	399.80 ± 53.80	376.20 ± 57.30
Control	147.60 ± 9.31	153.62 ± 11.24	7.24 ± 0.49	7.58 ± 0.50	7.42 ± 0.51	7.94 ± 0.58	373.20 ± 64.11	362.00 ± 59.96

Note: F is female, M is male.

**TABLE 8:** Effect of MPTA on clinical chemistry indices in rats.

Groups	Clinical chemistry parameters															
	ALB (mmol/L)		ALT (U/L)		AST (U/L)		BUN (mmol/L)		TCH (mmol/L)		CR (μmol/L)		GLU (mmol/L)		TP (G/L)	
	F	M	F	M	F	M	F	M	F	M	F	M	F	M	F	M
45 days																
48.20 mg/kg	27.44 ± 5.37	32.56 ± 5.28	37.75 ± 4.38	32.60 ± 5.15	192.77 ± 18.76	178.92 ± 17.68	7.83 ± 1.00	9.01 ± 0.31	1.40 ± 0.39	1.61 ± 0.37	83.54 ± 11.14	87.77 ± 15.20	8.07 ± 0.70	8.67 ± 0.79	65.71 ± 8.10	75.93 ± 4.58
9.64 mg/kg	28.51 ± 6.51	33.51 ± 6.52	29.84 ± 8.03	31.84 ± 8.84	192.72 ± 19.94	178.80 ± 20.33	8.76 ± 0.72	9.88 ± 0.95	1.21 ± 0.47	1.59 ± 0.43	76.59 ± 6.00	81.64 ± 4.65	8.03 ± 0.96	8.56 ± 0.83	61.67 ± 4.09	75.27 ± 4.27
1.93 mg/kg	30.64 ± 2.23	34.05 ± 5.24	29.99 ± 4.06	31.66 ± 7.81	191.23 ± 20.52	181.28 ± 15.48	8.34 ± 0.95	9.57 ± 0.69	1.39 ± 0.34	1.68 ± 0.30	75.87 ± 6.44	80.88 ± 5.87	8.43 ± 0.42	8.57 ± 1.06	62.55 ± 4.41	76.18 ± 3.74
Control	30.96 ± 2.39	33.52 ± 6.38	28.55 ± 3.39	31.88 ± 6.04	193.88 ± 19.13	183.61 ± 20.95	8.22 ± 0.66	8.74 ± 0.82	1.48 ± 0.48	1.71 ± 0.37	75.95 ± 5.88	79.79 ± 1.94	8.27 ± 1.07	8.65 ± 0.68	62.68 ± 3.18	76.03 ± 4.52
90 days																
48.20 mg/kg	30.07 ± 3.82	36.92 ± 2.41	34.77 ± 3.16	46.42 ± 11.09	177.05 ± 25.49	180.96 ± 25.46	6.63 ± 1.10	7.23 ± 0.79	1.11 ± 0.39	1.19 ± 0.62	74.17 ± 4.55	84.32 ± 4.12	8.15 ± 0.76	8.29 ± 0.57	68.00 ± 5.33	72.80 ± 6.27
9.64 mg/kg	31.30 ± 5.08	40.98 ± 5.11	32.90 ± 5.09	37.93 ± 4.97	177.22 ± 25.55	181.17 ± 25.30	6.15 ± 0.90	7.06 ± 0.62	1.43 ± 0.45	1.47 ± 0.38	72.63 ± 5.19	82.71 ± 5.74	8.22 ± 0.76	8.62 ± 0.73	68.64 ± 6.26	73.64 ± 5.03
1.93 mg/kg	31.33 ± 5.06	40.05 ± 4.23	32.73 ± 5.10	38.53 ± 3.87	177.03 ± 25.26	185.27 ± 21.58	6.22 ± 0.57	6.98 ± 0.85	1.46 ± 0.38	1.70 ± 0.49	73.28 ± 6.40	80.93 ± 3.87	8.20 ± 0.96	8.56 ± 0.67	68.08 ± 4.79	75.66 ± 4.45
Control	31.38 ± 5.03	40.93 ± 5.13	32.03 ± 2.75	37.70 ± 5.62	168.19 ± 25.32	181.08 ± 25.38	7.71 ± 0.75	7.33 ± 0.68	1.43 ± 0.42	1.52 ± 0.38	92.13 ± 6.09	83.23 ± 5.34	6.53 ± 1.10	8.55 ± 0.83	68.08 ± 4.79	73.04 ± 5.09
135 days																
48.20 mg/kg	36.42 ± 6.43	39.68 ± 6.31	35.79 ± 8.67	42.03 ± 8.29	153.13 ± 19.70	171.38 ± 19.66	6.74 ± 0.48	6.85 ± 0.96	1.28 ± 0.57	2.45 ± 0.19	85.63 ± 6.58	93.58 ± 5.11	7.31 ± 0.82	8.36 ± 0.62	70.05 ± 3.49	83.07 ± 4.45
9.64 mg/kg	36.51 ± 6.63	39.62 ± 6.44	35.89 ± 8.61	41.96 ± 8.59	153.32 ± 15.47	171.33 ± 19.44	6.85 ± 0.86	6.85 ± 0.86	1.35 ± 0.44	2.43 ± 0.30	84.93 ± 5.79	93.68 ± 5.47	7.30 ± 0.72	8.48 ± 0.57	70.30 ± 2.72	83.11 ± 4.23
1.93 mg/kg	36.64 ± 6.63	41.63 ± 2.20	35.93 ± 9.01	42.49 ± 8.18	153.27 ± 19.70	179.26 ± 15.06	6.94 ± 0.52	6.94 ± 0.52	1.34 ± 0.55	2.36 ± 0.40	85.08 ± 7.21	93.83 ± 5.11	7.27 ± 0.49	8.01 ± 0.75	70.67 ± 4.74	80.87 ± 6.26
Control	36.39 ± 4.04	39.55 ± 6.31	32.03 ± 2.75	43.12 ± 9.15	157.39 ± 14.87	171.28 ± 19.58	7.03 ± 0.75	7.03 ± 0.75	1.42 ± 0.57	2.25 ± 0.17	83.36 ± 4.55	94.23 ± 4.63	7.30 ± 0.75	8.32 ± 0.79	70.95 ± 3.95	82.55 ± 4.20
180 days																
48.20 mg/kg	40.90 ± 5.15	34.42 ± 5.05	38.00 ± 5.21	33.80 ± 5.23	168.20 ± 25.54	186.85 ± 25.55	7.34 ± 0.69	7.48 ± 0.66	1.93 ± 0.49	1.85 ± 0.51	90.68 ± 5.17	80.93 ± 5.44	6.70 ± 0.83	7.27 ± 0.71	77.68 ± 5.35	71.50 ± 5.44
9.64 mg/kg	40.90 ± 5.05	33.05 ± 4.41	37.90 ± 5.16	35.63 ± 3.47	168.30 ± 25.76	188.46 ± 29.22	7.42 ± 0.93	7.29 ± 0.92	1.90 ± 0.71	1.78 ± 0.63	90.66 ± 4.51	82.00 ± 6.17	6.59 ± 1.13	7.43 ± 1.10	77.68 ± 5.53	69.71 ± 2.64
1.93 mg/kg	39.68 ± 3.90	30.89 ± 3.08	33.08 ± 4.97	35.84 ± 3.1	166.95 ± 23.38	175.95 ± 9.99	7.33 ± 0.55	7.00 ± 0.39	1.74 ± 0.62	1.82 ± 0.51	88.83 ± 1.59	79.43 ± 4.06	6.76 ± 0.73	7.45 ± 0.39	75.56 ± 3.79	70.16 ± 5.51
Control	40.75 ± 4.98	34.45 ± 5.01	37.10 ± 5.14	33.93 ± 4.87	168.19 ± 25.10	160.55 ± 20.69	7.71 ± 0.75	7.43 ± 0.71	1.71 ± 0.44	1.78 ± 0.42	92.13 ± 6.09	34.56 ± 5.10	6.53 ± 1.10	7.22 ± 0.80	76.24 ± 3.69	71.66 ± 5.44

Note: F is female, M is male.

**TABLE 9:** Effect of MPTA on organ coefficients in rats.

Groups	Organ coefficient (%)													
	Liver		Kidney		Spleen		Stomach and intestines		Lung		Heart		Testicles	Ovaries
	F	M	F	M	F	M	F	M	F	M	F	M		
48.20 mg/kg	3.52 ± 0.25	2.58 ± 0.15	0.69 ± 0.05	0.60 ± 0.03	0.20 ± 0.02	0.16 ± 0.00	9.38 ± 0.99	7.28 ± 0.33	0.54 ± 0.05	0.47 ± 0.04	0.38 ± 0.01	0.32 ± 0.02	0.59 ± 0.03	0.04 ± 0.01
9.64 mg/kg	3.79 ± 0.18	2.47 ± 0.12	0.74 ± 0.04	0.57 ± 0.03	0.20 ± 0.01	0.15 ± 0.01	9.36 ± 0.67	6.94 ± 0.31	0.53 ± 0.05	0.44 ± 0.04	0.35 ± 0.02	0.32 ± 0.01	0.55 ± 0.04	0.04 ± 0.00
1.93 mg/kg	3.80 ± 0.23	2.51 ± 0.17	0.74 ± 0.05	0.58 ± 0.05	0.20 ± 0.01	0.16 ± 0.01	9.36 ± 0.58	7.05 ± 0.63	0.54 ± 0.07	0.46 ± 0.02	0.39 ± 0.03	0.33 ± 0.02	0.57 ± 0.02	0.04 ± 0.00
Control	3.50 ± 0.36	2.45 ± 0.14	0.68 ± 0.06	0.56 ± 0.04	0.19 ± 0.02	0.15 ± 0.01	8.85 ± 0.48	6.90 ± 0.48	0.50 ± 0.03	0.43 ± 0.04	0.37 ± 0.02	0.32 ± 0.02	0.56 ± 0.04	0.04 ± 0.00

Note: F is female, M is male. Organ coefficient = (organ weight/body weight) × 100%.

## Discussion

PAs have been widely used due to their good pharmacological activity (Xiao et al., 2007; Bae et al., 2012; Sreenivasmurthy et al., 2022), but scientific evaluation studies on their toxicity and side effects are lacking. A complete and systematic safety evaluation is essential for the safety of clinical drug use. Except for the previous oral acute toxicity and 90-days repeated dosing toxicity studies of MPTA in rats (Dong et al., 2022a), no further risk assessment of MPTA has been reported. In this study, oral acute toxicity test in mice, *in vivo* genotoxicity test, teratogenicity test in rats and chronic toxicity test were conducted to further evaluate the possible toxicity risk of MPTA during use and to provide data support for subsequent residue evaluation.

The oral LD_50_ of MPTA was 481.99 mg/kg in rats (Dong et al., 2022a), and since the dose selection for the *in vivo* genotoxicity test carried out in this study was based on mice, the oral acute toxicity of MPTA to ICR mice was carried out, and coincidentally, the LD_50_ in mice was also 481.99 mg/kg, and according to the chemical toxicity classification criteria, MPTA is According to the chemical toxicity classification, MPTA is of moderate toxicity (51–500 mg/kg). The previously reported effective dose of 5.08 mg/kg for MPTA to exert anti-inflammatory effects is considered to have a manageable safety distance, although no clear safety range has been obtained.

MPTA has good properties for use as a potential anti-inflammatory agent, but a large number of toxicities and adverse effects have been reported for nonsteroidal anti-inflammatory drugs (NSAIDs) in clinical practice, such as irritation of the gastrointestinal mucosa (Melarange et al., 1994), biochemical abnormalities due to mild liver damage (Portmann et al., 1975; Ali et al., 2011), hematopoietic dysfunction (Canada and Burka, 1968; Wiesen et al., 2009), interstitial nephritis due to prolonged oral administration (Lucas et al., 2019; Klomjit and Ungprasert, 2022), bleeding in delivery (aspirin) (Hastie et al., 2021) and fetal malformations (indomethacin) (Klein et al., 1981; Sangem et al., 2008). Therefore, this study focused on the long-term toxicity, genotoxicity and toxicity to reproductive development of MPTA. Hematological tests are often used to assess the physiological and pathological status of the body. Parameters such as WBC, RBC, HGB and PLT can reflect the inflammatory status of the body and the effect of drugs on the hematopoietic system. In chronic toxicity tests, MPTA at the doses tested (48.20–1.93 mg/kg) did not produce significant effects on hematological parameters in rats. The effects on liver and kidney are often reflected by clinical chemistry, as liver injury due to the drug often causes concomitant increases in ALT and AST, while kidney injury also causes increases in serum parameters such as BUN and CR. In this test, MPTA did not have a significant effect on clinical chemistry parameters in the rats at the doses tested. The organ coefficients can be used to initially locate the toxic target organs of the drug, and there were no significant differences between the dose groups of MPTA and the control group in this study. Histopathological examination is considered the “gold standard” for disease/toxic injury diagnosis (Karadag Soylu, 2020), and in further histopathological examination, the highest dose of 48.20 mg/kg MPTA did not cause significant toxic lesions in major organs susceptible to NSAIDs, such as liver, kidney, and intestine. Therefore, it can be concluded that MPTA lacks the risk of chronic toxicity.

Genotoxicity studies are an important part of drug entry into clinical trials and marketing, and are closely related to other studies, especially carcinogenicity and reproductive toxicity (Ellinger-Ziegelbauer et al., 2009). In the three *in vivo* genetic mutation tests (sperm abnormality test, micronucleus test and chromosomal aberration test) conducted in this study, all test results were negative at the doses tested (60.25–241.00 mg/kg) and therefore MPTA was not considered genotoxic. In the next teratogenic test in rats, we found that body weight gain was inhibited in females at the tested doses of 30.12 mg/kg and 120.50 mg/kg and that embryonic uptake and fetal malformation rates were increased compared to the negative control group. In another *in vitro* study, California poppy extracts containing PRO showed no inhibitory effect in placental cells (BeWo b30) and the comet assay did not show genotoxic potential. However, as data on the pharmacokinetic profile of PRO are lacking, it cannot be demonstrated that the concentrations tested *in vitro* can achieve sufficient *in vivo* effects (Spiess et al., 2022b). In addition, it has been shown that an increase in 5-Hydroxytryptamine leads to an inhibition of weight gain in pregnant mice and reduces maternal estrogen and progesterone levels leading to early fetal developmental disorders (Han et al., 2022). It is possible that the potential embryonic developmental toxicity of MPTA also affects the levels of estrogen in rats. The results of another study also showed that PRO can be transferred from the mother to the fetal circuit and reach homeostasis through the placental barrier (Spiess et al., 2022a). This also provides a partial explanation for the potential embryotoxicity risk of PRO, but the cause of embryotoxicity due to MPTA and its safety for target animals should still be further investigated.

It is worth noting that MPTA is a mixture and its toxicity results can only be representative of the test substance itself, as the toxic effects of the mixture depend on the composition of the substances in the mixture, the proportions, and the interactions between the compounds (independent, synergistic, additive, or antagonistic) (Bart et al., 2022). Therefore, the main active substance in MPTA should be further tested when used alone.

## Conclusion

The oral LD_50_ of MPTA was 480 mg/kg. In a test of long-term repeated dosing, no signs of potential toxicity were shown at different doses. It lacks mutagenicity, but there may be reproductive toxicity or embryonic developmental toxicity at dosage levels above 7.53 mg/kg. This study suggests that MPTA could be used for further drug development studies. Detailed in-depth studies will continue afterwards to address the teratogenicity results.

## Data Availability

The original contributions presented in the study are included in the article/supplementary material, further inquiries can be directed to the corresponding authors.
